# Sudden Death of a Young Hemophiliac by Low-Velocity Blunt Knee Trauma in Bullock Cart Run-Over Fatality

**DOI:** 10.7759/cureus.12623

**Published:** 2021-01-11

**Authors:** Mohit Chauhan, Chittaranjan Behera, Ashish Rustagi

**Affiliations:** 1 Forensic Medicine, Government Medical College & Hospital, Chandigarh, IND; 2 Forensic Medicine, All India Institute of Medical Sciences, New Delhi, IND; 3 Orthopaedics, Vardhman Mahavir Medical College and Safdarjung Hospital, New Delhi, IND

**Keywords:** popliteal injury, knee trauma, sudden death, hemophilia, bullock cart fatality

## Abstract

A young bullock cart driver was pushing bulls hard in stunt and frolic. The cart sped up and he lost control and toppled in front of the iron wheel, which ran over his lower limb around the knee. Concomitant hemophilia further complicated the popliteal artery bleed, and the patient succumbed within hours of injury, despite medical aid. Sudden death is rare in congenital or acquired hemophilia. Popliteal artery injuries usually threaten the limb in high-velocity blunt or penetrating trauma in comparison to other peripheral arteries. However, fatality after popliteal artery injury in low-velocity blunt trauma is rare. Bullock cart is a very slow mode of transport. But animals can show unpredictable and aggressive behavior when driven in carts, which poses considerable risk of fatality to driver and occupants if they sustain vascular or regional injuries. As there is scarce literature about bullock cart-related injuries, this paper focuses on bullock cart run-over fatalities and sudden death in young hemophiliacs.

## Introduction

Popliteal artery is the main vascular supply of the knee and leg. It is juxtaposed to the femur, tibia, and knee joint and is in a “tethered” position behind the knee and between the adductor hiatus proximally and the soleal arch distally. This makes the vessel relatively fixed, and it rarely escapes injury in high-velocity blunt or penetrating trauma, for example, tibial or supracondylar fracture dislocation, gunshot, or stabbing of the leg and knee [1]. Its transection, laceration, perforation, fistula, or intimal tear is seen in 28%-46% of blunt trauma to the legs [1].

Synovial vessels bleed badly after injury in hemophiliacs due to altered coagulation. The synovium gets thickened by default iron absorption in these bleeds because of physiological function. Angiogenesis in thickened synovial lining potentiates further bleeds forming a vicious cycle. The elbows, ankles, and knees are commonly affected joints in hemophiliacs [2].

Arterial injury in hemophiliacs is difficult to treat owing to inadequate coagulation combined with bony deformities due to alternate bleeding and fibrosis [3]. Increased probability of limb salvage is possible with early recognition and advanced vascular repair. High-velocity blunt trauma to the knee may vitiate popliteal artery injury leading to amputation; however, death is quite unusual.

Poor roads, low economic status of farmers, unaffordable running, and maintenance cost of motorized vehicles are factors making bullock carts socially and culturally the most acceptable mode of transportation in developing countries. Bullock cart races are also organized as sporting events in different parts of the world. However, speed control is a problem in these animal-driven vehicles. This sometimes requires constrained or reflex application of unscientific and dangerous braking methods, putting the life of the rider or occupants in danger.

## Case presentation

**Figure 1:**
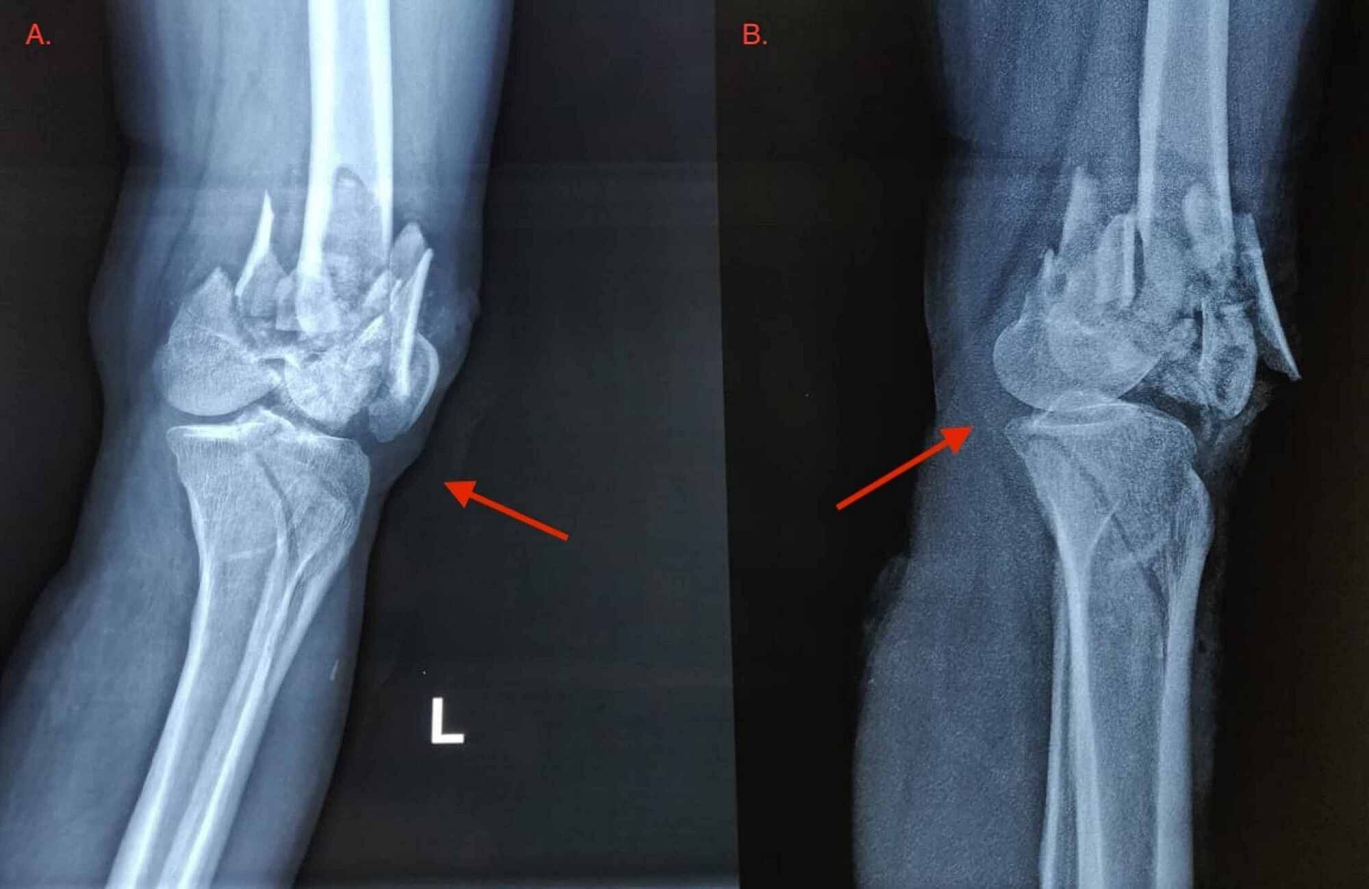
X-ray of the left knee with the leg. (A) Anteroposterior and (B) lateral views showing comminuted intercondylar fracture femur, fracture patella, and fracture proximal tibia (red arrows).

This case presents an alleged history of sustaining run-over injury to the left thigh by the iron wheel of a bullock cart driven by a 21-year-old young hemophiliac, with no other medical comorbidities. A splint was applied and he was immediately referred to a higher center for expert management. Factor VIII value of 3.44% with no autoantibodies revealed him to be a moderate hemophiliac-A. The patient presented in a hypovolemic shock, with swelling and deformity of the left distal thigh and knee, with absent ipsilateral distal pulsations. Doppler of the left lower limb revealed turbulence at the popliteal artery with distal monophasic flow. The case was diagnosed as a closed comminuted distal femoral fracture and patella fracture with proximal tibial fracture of the left side (Figure 1) with popliteal artery, for which a spanning external fixator was applied and popliteal artery exploration and repair was done at a tertiary care center.

Factor VIII concentrate and blood was infused owing to falling hemoglobin and hematocrit levels. However, the patient succumbed to his injuries within 24 hours.

**Figure 2:**
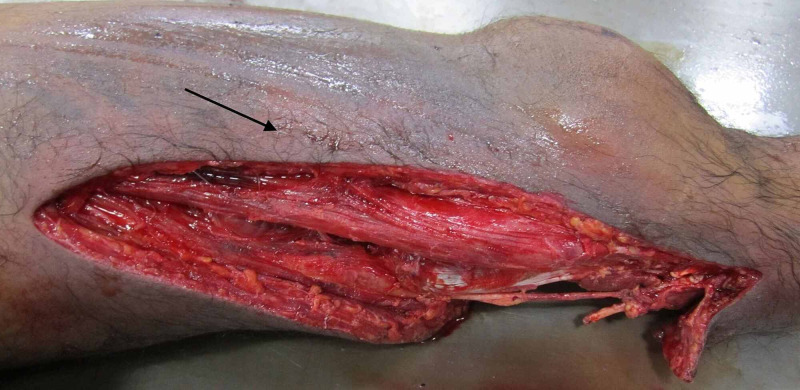
Extensive bruising of the left lower limb over the knee and superolateral aspect of the thigh.

The external fixator was removed before autopsy, and extensive bruising of the left lower limb over the knee and superolateral aspect of the thigh was seen (Figure 2).

**Figure 3:**
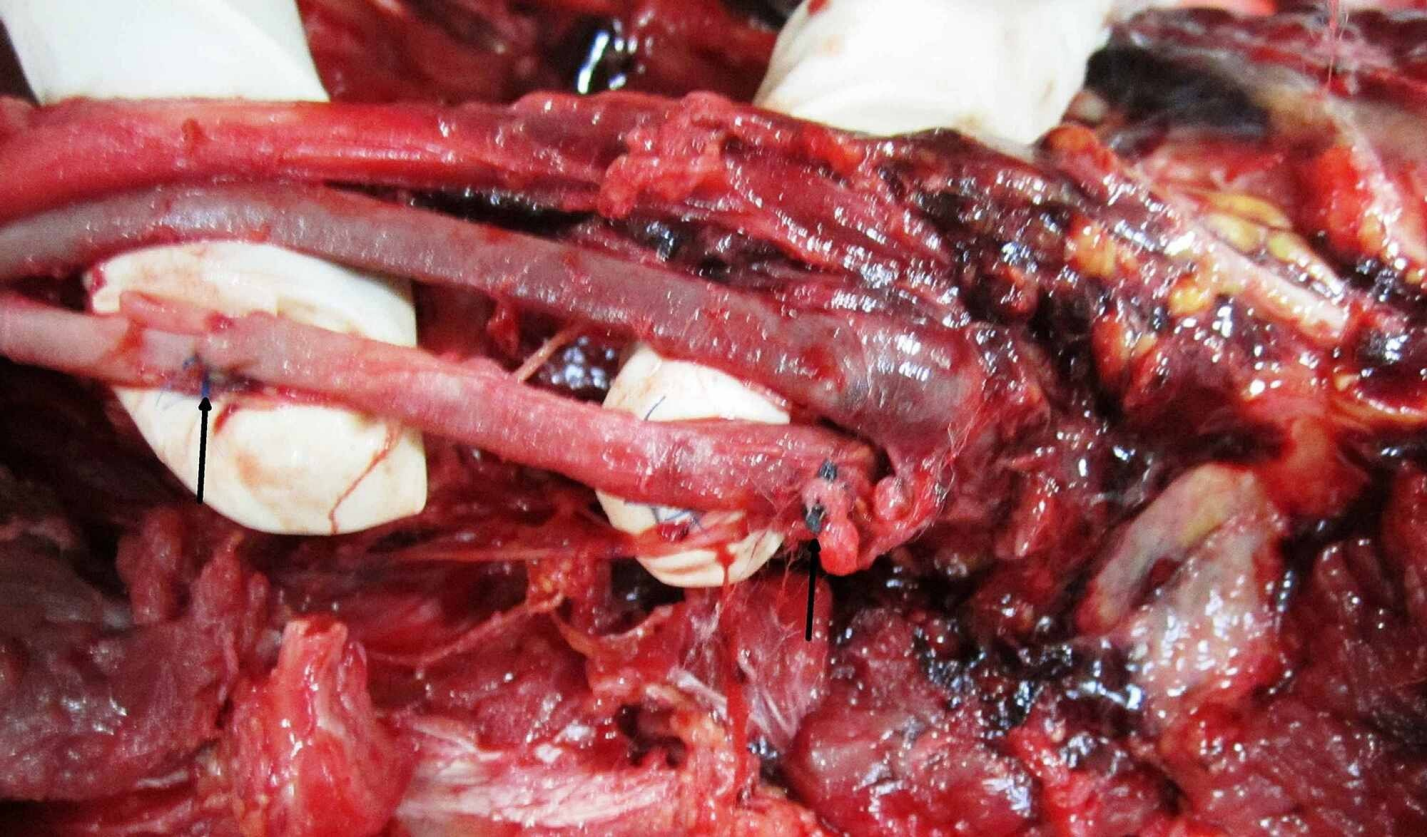
Polypropylene 6-0 interrupted sutures over the anterior wall at the distal end of the popliteal artery in upper third just before bifurcation (black arrows).

Stitch removal at the popliteal fossa showed extensive effusion in both heads of gastrocnemius and tissues. Polypropylene 6-0 interrupted sutures were present over the anterior wall at the distal end of the popliteal artery in upper third just before bifurcation. The removal revealed corresponding rents of size 0.4 × 0.3 cm and 0.7 × 0.5 cm (Figure 3).

Internal organs were pale. The spleen was enlarged. Death occurred due to hemorrhagic shock upon blunt force trauma to the popliteal artery in a hemophiliac.

## Discussion

Sports, traffic, or falls cause disruptions and occlusions of the popliteal artery posing risk to the limbs, which become more frequent with age with a male predominance owing to more outdoor pursuits [4]. Vascular complications after trauma to the knee are rare but need early recognition and treatment to avoid significant morbidity and medicolegal ramifications. Tears of intima and media prevail over complete thrombosis of the popliteal artery in blunt trauma [5]. The popliteal artery is injured in 19% of all extremity arterial injuries ranging 20%-26% and 20%-75% in military and civilian population, respectively [6]. Blunt knee trauma has a tenfold risk of amputation than that of penetrating trauma, if both involve the popliteal artery, due to less striking presentation and lower index of suspicion in the former [7].

Clinching cases of popliteal artery injury complicating amputation following blunt trauma knee without dislocations [8] or limb salvage with dislocation due to outcome based on multiple factors have been reported. However, death following popliteal artery injury in blunt trauma is rare [9]. Reid et al. [10] reported death of an adolescent due to delayed presentation on the third day after blunt knee sports injury with development of systemic inflammatory illness due to popliteal artery occlusion. Lang et al. [11] evaluated concomitant injuries, complications, amputation rates, and outcomes in 64 cases of blunt (54.7%) and penetrating (45.3%) lower limb trauma with popliteal artery injury, along with a death. Wagner et al. [12] evaluated 100 consecutive cases of blunt popliteal artery trauma and reported mortality rate of 3% with three deaths due to associated or supervening factors, with none directly caused due to the arterial injury.

Mortality is associated with penetration causing early hemorrhagic shock from the injured proximal arterial segment. In contrast, early limb loss is more common with blunt distal vascular injury of popliteal and tibial arteries. However, hemophilia complicating death following blunt injury due to fracture of the knee joint with associated popliteal injury is quite rare and has not been reported before. A hemophiliac road traffic accident victim died on the sixth day due to head injury and retroperitoneal hematoma despite successful limb revascularization for blunt trauma to the knee [13].

The bullock cart is a relatively slow moving vehicle. There is astonishingly scant literature describing bullock cart-related accidental strangulation [14], impact [15], or injuries [16]. Hence, news reports have been reviewed in addition to standard literature to present a comprehensive overview of fatal and nonfatal, exclusive run-over injuries by bullock carts.

A three-year-old boy accompanying his father on a bullock cart slipped and was crushed to death after being run over by a cart wheel [17]. A rare case of surviving run over by a bullock cart wheel has been reported where an inebriated male survived three and two run overs of the head and legs after he fell on the track during a bullock cart race festival [18]. Another case of run over has been reported where the animal after being whipped to run in a bullock cart race got amok to head toward one of the persons and injured his foot by run over [19]. In another case, a school child riding a bicycle in the opposite direction got his shirt entangled in the horns of one of the animals and was run over at his neck by the cart moving on the road with nine other carts, which were all carrying sand [20].

The hemophiliac patient sustaining blunt trauma succumbed to hemorrhagic shock owing to popliteal artery injury, despite optimum surgical management instituted on time. Catastrophic bleeding through any other viscera, especially an enlarged spleen, though present in this case, was ruled out. Thrombosis, otherwise less common in comparison to injuries of intima and media in hemophiliacs vis-à-vis the normal population, would have substituted death with amputation by default in the present case if the patient would not have been a hemophiliac. Similar blunt trauma with coexistent hemophilia causing sudden unexpected death has not been reported. The mode and setting of sustaining trauma in low-velocity bullock cart vehicular accident is highly unusual.

## Conclusions

Surgeons need to have a high index of suspicion to diagnose arterial injury and bleeding disorder unless proven otherwise in blunt lower extremity trauma. A simple, fast, and inexpensive test to estimate bleeding and clotting time can rule out various coagulopathies and save lives. Because award of punishment may become complicated in sudden unexpected deaths in hemophiliacs, medicolegal opinion should be furnished in light of a thorough review of clinical records. Forensic pathologists carry the utmost responsibility to explain aggravation of the nature of injury sustained either accidentally or intentionally in those with pre-existing conditions.
